# Breast Abcess and Tuberculosis and its Diagnostic Challenges: A Two-Year Prospective Study in Karachi, Pakistan

**DOI:** 10.7759/cureus.5909

**Published:** 2019-10-15

**Authors:** Batool Zehra Asjad, Mir Arsalan Ali, Bushra K Naeem, Mehmood Khan, Urooj Ahmed Abbasi, Zakia Nehal, Sara Siddiqui

**Affiliations:** 1 Surgery, Hamdard University Hospital, Karachi, PAK; 2 Surgery, Ziauddin University, Karachi, PAK; 3 General Surgery, Jinnah Post Graduate Medical College, Karachi, PAK; 4 Surgery, Jinnah Postgraduate Medical Center, Karachi, PAK; 5 Surgery, Jinnah Postgraduate Medical Centre, Karachi, PAK

**Keywords:** breast abcess, tuberculosis, lactation, extrapulmonary tuberculosis

## Abstract

Breast tuberculosis (TB) is rare among extrapulmonary tuberculosis cases, and the diagnosis is usually preceded by a high index of suspicion and findings of granulomatous lesions. We conducted this study to evaluate different clinical presentations of breast TB, including its diagnosis and association with lactation. We also examined the association of breast TB with TB elsewhere in the body and contact history. This prospective, descriptive study was conducted at a tertiary care hospital in Karachi, Pakistan from March 2017 to March 2019. The study population consisted of 100 women of age ranging from 30 to 39 years after 10 patients were lost to follow-up. After providing informed written consent and a histopathology or culture report, participants completed a proforma. We used IBM SPSS Statistics for Windows, Version 22.0 (IBM Corp., Armonk, NY) to analyze the data, which were kept confidential. Twenty-four patients had diabetes, while 36 patients had no comorbidities. The most consistent symptoms were breast pain (98%), breast lump (89%), fever (83%), and discharge (45%). Of the 100 women, 34 were diagnosed with TB, while 66 women had nonspecific inflammation. Sixty-nine women were lactating, and 31 were nonlactating. Of the 69 lactating women, 21 had been diagnosed with TB while 48 had nonspecific inflammation. TB represents a great burden in underdeveloped countries like Pakistan. Therefore, concerned departments must take necessary action to facilitate early diagnosis and prompt treatment.

## Introduction

Tuberculosis (TB) is the found to be one of the persistent human infections and widespread all around the world [1]. It has been well known as a pulmonary disorder but 17.5 % cases also have extrapulmonary manifestation [2]. Breast tuberculosis has been a rare entity constituting only 0.1% of extrapulmonary tuberculosis [2]. However, in developing countries it accounts for about 3.0% of breast diseases which were treated surgically [3]. The clinical features of breast tuberculosis vary and lesions may be confused with breast carcinoma or pyogenic abscess [4]. The standard diagnostic methods for the identification and confirmation of Mycobacterium tuberculosis is made either by Ziehl-Neelsen staining or culturing. However, these methods have low sensitivity [4]. Therefore, it is hard to diagnose tuberculosis of the breast and in order to have a definitive diagnosis patients usually undergo numerous investigations and ineffective treatments [4]. Other methods like surgical biopsy and fine needle aspiration cytology (FNAC) may also be inconclusive [5]. In most of the cases, high level of suspicion is required for the diagnosis of breast tuberculosis, which includes presence of langhans giant cells in granulomatous lesion, Mycobacterium tuberculosis in culture and response to antitubercular therapy (ATT) [5]. The objective of the present study is to assess different clinical presentation and diagnosis of breast tuberculosis, it’s association with lactation and relation with tuberculosis elsewhere in body or contact history.

## Materials and methods

This prospective, descriptive type of study was conducted at Hamdard University Hospital Karachi, Pakistan from March 2017 to March 2019. Female patients presenting with breast pain or lump, with or without fever or discharge were included in the study after providing written informed consent. Patients previously operated for breast diseases were excluded from the study population. The study protocol was approved by hospital ethics committee. Data were collected via patient-completed proformas. We also collected data on age, comorbidities, symptoms (such as breast pain, breast lump, fever, and discharge), lactating status, histological analysis, TB contact history, and TB presence elsewhere in the body. Patient data was kept confidential, and IBM SPSS Statistics for Windows, Version 22.0 (IBM Corp., Armonk, NY) was used for data analysis.

## Results

One hundred ten women were initially surveyed; however, 10 patients were lost to follow-up, leaving a total of 100 participants in our study. The patients' age range was 30 to 39 years. Twenty-four patients had diabetes, 36 patients had no comorbidities. Thirty-four patients had been diagnosed with TB, while 66 patients had nonspecific inflammation. Sixty-nine women were lactating, and 31 patients were nonlactating. Of 69 lactating patients, 21 had been diagnosed with TB while 48 patients had nonspecific inflammation. Of the 31 nonlactating patients, 13 were diagnosed with TB while 18 patients had nonspecific inflammation (Table 1).

**Table 1:** Lactation and tuberculosis

	Tuberculosis	Non-Specific Inflammation	Total patients
Lactating	21	48	69
Non-Lactating	13	18	31
Total patients	34	66	100

Twenty-two patients had a history of TB contact, and of those, 14 patients had breast TB (Table 2).

 

**Table 2:** TB contact history and breast TB

	Tuberculous inflammation	Nonspecific inflammation	Total patients
TB contact +ve	14	8	22
TB contact -ve	20	58	78
Total patients	34	66	100

The most common symptoms were breast pain (98%; Table 3), breast lump (89%), fever (83%), and discharge (45%).

**Table 3:** Breast pain and TB

	Tuberculous inflammation	Nonspecific inflammation	Total Patients
Breast pain present	33	65	98
Breast pain absent	0	2	2
Total patients	33	67	100

## Discussion

In the underdeveloped world, breast TB accounts for fewer than 1% of the total breast disease incidence [1]. Breast TB is rare in extrapulmonary TB cases. Baharoon et al reported that in 1829, Sir Astley Cooper reported tuberculous mastitis as the pioneer case and named it “bosom’ s scrofulous swelling” [1]. Breast TB can be primary (i.e., absent in a different area of the body) or secondary (i.e., present elsewhere in the body), which is somewhat more common than primary [4,6]. Primary breast TB can spread either by abrasions or openings present at the ductules of the nipple [4]. Mirsaeidi et al. reported that the age of patients with breast TB ranges from 20 to 40 years, which was similar to our study range of 30 to 39 years [2]. One study found that in a large number of patients, most breast lesions were abscesses and skin fistulations, most of which were found in young women [3]. Breast TB is more common in women, very rarely involving men. However, a recent study found six of 160 breast TB patients were men [7]. TB is known to spread through contact. In a study by Sreeramareddy et al., 11% of patients with a history of contact with a known TB case eventually developed extrapulmonary TB [8]. In our study, 22 patients had a history of TB contact, and of those, 14 patients had breast TB. Lactation can escalate the vulnerability of the patient for breast TB; 33% of the women with breast TB in one study were lactating, and in our study, 69% of the women were lactating [9]. The increased vulnerability from lactation may be due to trauma to the ductules and increased blood supply to the tissue common during lactation, making the tissue more prone to inflammation [4,7]. When the TB lesion is irregular, stony hard, and adherent to the skin and underlying structures, it becomes difficult to differentiate this condition from breast cancer [1]. In Ocal et al.’s study, all patients had unilateral involvement of breast TB, which is consistent with our findings. Bilateral involvement of the breast is very infrequent, and according to our results, both breasts have equal tendencies to develop TB [10]. The diagnosis of TB is difficult because the typical findings of matted lymph nodes, several sinuses, ulcers, and breast swelling are absent [5]. The specific investigation for breast TB is Ziehl Neelsen stain or culture [11]. Of the diagnosed cases of breast TB, 12% are branded with acid-fast, and 25% are positive for TB bacilli [11]. Many authors consider FNAC as an accurate means of breast TB diagnosis. One study reported 73% of cases with caseous necrosis and epithelioid giant cells [3]. In another study, red hot tender nodularity of the breast along with the pathological findings of FNAC yielded 100% accuracy in diagnosing TB, whereas fat necrosis, plasma cell mastitis, and actinomycosis (which resemble the pathological results of breast TB) were not reliable factors for differentiating breast TB from other pathologies [7].

Our study was limited in that no staining tests (e.g., Zehl Nelson or immunohistochemistry) were performed on breast tissue samples due to limited resources. Future studies should involve staining all breast tissue samples with immunohistochemistry by anti-Mycobacterium bovis bacille Calmette-Guérin polyclonal antiserum to improve the diagnostic accuracy in breast TB [12].

## Conclusions

Breast abscess is a very rare presentation of TB. Identifying symptoms and using histopathological examinations early is ideal for diagnosing breast TB. In cases with a high index of suspicion for TB, tissue biopsy should be sent at the time of surgery. TB represents a great burden in underdeveloped countries like Pakistan. Therefore, concerned departments must take necessary action to facilitate early diagnosis and prompt treatment.
